# The nurse teacher’s pedagogical cooperation with students, the clinical learning environment and supervision in clinical practicum: a European cross-sectional study of graduating nursing students

**DOI:** 10.1186/s12909-022-03445-0

**Published:** 2022-06-28

**Authors:** Camilla Strandell-Laine, Leena Salminen, Katrín Blöndal, Pilar Fuster, Susan Hourican, Sanna Koskinen, Helena Leino-Kilpi, Eliisa Löyttyniemi, Juliane Stubner, Marija Truš, Arja Suikkala

**Affiliations:** 1 Department of Nursing Science, University of Turku, 20014 Turku, Finland; 2 Novia University of Applied Sciences, Turku, Finland; 3 Lovisenberg Diaconal University College, Oslo, Norway; 4 Turku University Hospital, Turku, Finland; 5 Surgical Services Landspitali – The National University Hospital of Iceland, Reykjavik, Iceland; 6 Faculty of Nursing, University of Iceland, Reykjavik, Iceland; 7 Department of Nursing, Faculty of Medicine and Health Sciences, Universitat Internacional de Catalunya, Barcelona, Spain; 8 School of Nursing, Psychotherapy & Community, Dublin City University, Dublin, Ireland; 9 Department of Biostatistics, University of Turku, Turku, Finland; 10 Martin Luther University Halle-Wittenberg, Halle (Saale), Germany; 11 Department of Nursing, Faculty of Health Sciences, Klaipeda University, Klaipėda, Lithuania; 12 Diaconia University of Applied Sciences, Helsinki, Finland

**Keywords:** Clinical learning environment, Cross-sectional study, Final clinical practicum, Graduating nursing student, Mentor, Nurse teacher, Supervision

## Abstract

**Background:**

A supportive clinical practicum experience may enhance the successful transition and socialization to working life of graduating nursing students. Nurse teachers have the main responsibility of supporting and guiding nursing students with their pedagogical expertise during the students’ clinical practicum. Thus, the clinical role of nurse teachers is seen as an essential part of a high-quality clinical practicum. Nursing students appreciate the nurse teacher’s cooperation with students, but it is often reported to be unattainable. The aim of this study was to explore and compare graduating nursing students’ experiences of the nurse teacher’s pedagogical cooperation with students, the clinical learning environment and supervision in their final clinical practicum, and to analyze factors associated with these experiences in six European countries.

**Methods:**

A cross-sectional comparative international survey design was used. The modified Clinical Learning Environment, Supervision and Nurse Teacher (CLES+T) Scale, with a new subscale measuring the nurse teacher’s pedagogical cooperation with students, was used. A convenience sample of graduating nursing students in Finland, Germany, Iceland, Ireland, Lithuania and Spain completed the online survey in 2018–2019. The data were analyzed using a Chi-Square test, Pearson’s correlation coefficients, and linear models.

**Results:**

A total of 1796 (response rate 49%) nursing students completed the survey. Overall, students had positive experiences of the nurse teacher’s pedagogical cooperation, the clinical learning environment and supervision in their final clinical practicum. Students in Spain had the most positive experiences. Educational background factors appeared to be associated with the students’ experiences of the nurse teacher’s pedagogical cooperation with students, the clinical learning environment and supervision. The relationships between the subscale Nurse teacher’s pedagogical cooperation with students and the Clinical Learning Environment and Supervision Scale were perceived as weak to strong depending on the country.

**Conclusions:**

This study reveals that nurse teachers play an essential role in supporting and guiding nursing students’ final clinical practicum. In this light, researchers, educators, and leaders should collaborate seamlessly between educational institutions and healthcare organizations to establish the nurse teachers’ pedagogical cooperation role within the clinical learning environment.

## Background

The final clinical practicum experiences of graduating nursing students (hereafter GNSs) are found to be important for the development of the nursing identity, motivation for nursing as a career [1] and its successful start [1–4]. Nursing students’ clinical practicums are dispensed under the supervision of a mentor from the clinical practice and pedagogical cooperation of a nurse teacher (hereafter NT) from the educational institution [5, 6]. The pedagogical cooperation of an NT refers to the cooperation between the students and teacher during the clinical practicum, which aims to support students clinical learning [7]. Therefore, graduating students’ experiences of the NT’s pedagogical cooperation with students in promoting the clinical learning of students [7] (hereafter NT cooperation), the clinical learning environment (hereafter CLE) in terms of pedagogical atmosphere, leadership style of the ward manager and premises of care on the ward as well as mentor supervision in their final clinical practicum are essential contributors toward ensuring sufficiency and adequacy of the future nursing workforce [8, 9].

A supportive and safe CLE as well as socialization are seen as key elements for GNSs in their successful transition to working life as well as for young nurses in their first work environments [2, 10]. Raising the quality of nursing education, especially the quality and supportive elements of the CLE [11, 12], may also contribute toward higher student pass rates of nursing degree studies [13]. These conditions of the CLE place new demands on nursing education, particularly on NTs. Among students, NTs are valued as key persons in providing student learning evaluations, integrating theoretical and practical knowledge, helping students to understanding their roles as students, and providing emotional support during the clinical practicum [14, 15]. Thus, a high frequency of NT cooperation is seen to be associated with students’ positive clinical practicum experiences [16, 17]. By providing constructive feedback and supporting the students’ confidence and competence achievement, NTs may also strengthen the motivation of students to pursue nursing as a career [13, 18]. This study focuses on final clinical practicum experiences, with particular emphasis on NT cooperation from the perspective of GNSs.

As already stated in the European Union directive in 2005 [5], NTs have the main responsibility of the student supervision in clinical practicum [5, 15, 17], albeit the clinical role of NTs has been ill-defined over the years and varies across European countries and clinical placements [8, 19, 20]. However, European nursing students commonly appreciate the cooperation they have with their NTs [14, 17, 20–23], despite having reported a lack of support from NTs [14, 19, 21, 22, 24].

Supervising mentors i.e., assigned Registered Nurses (RNs) from the nursing students’ practicum ward, together with supervising NTs, play an important role in ensuring a supportive clinical practicum experience and promoting opportunities for the learning of students [18, 25]. As such, mentors are considered to be essential professional role models for students by supporting their professional development [26]. The mentor supervision relationship, commonly conducted as a named, individualized, one-to-one relationship, has been reported as being the most important component of a student’s clinical practicum experience [8, 18, 24, 27]. When comparing the group and one-to-one supervision models, the most satisfying supervision model for nursing students has been the one-to-one model [8, 27, 28]. Moreover, the high frequency of this mentor relationship [6, 24, 28] and private unscheduled mentor-student discussions [24] are seen to be associated with a student’s positive clinical practicum experience.

There are several student-related individual factors that may positively impact on the clinical practicum experiences of students. The first career choice when entering a nursing program [13, 29] and prior work experience in healthcare have been reported to be associated with good clinical practicum experiences [30]. Therefore, although a student’s higher study motivation [19], higher emotional intelligence [31], better clinical practicum experience and the reality of nursing practice play an important role toward the completion of studies and career intent [13, 29], there remains a lack of evidence in these areas [11].

Despite the Bologna process [32] and the requirements for clinical practicum [5, 33], and the common labor market in Europe, there are repeatedly reported disparities in educational factors, such as the implementations of the clinical practicum, i.e. the length and amount of clinical practicum periods [6, 19, 34–36]. However, a short or perfunctory clinical practicum may not enhance the socialization of students to the practicum placement [37]. Therefore, a long clinical practicum [6, 24, 34, 38] and high-quality theoretical pre-practicum studies [19] are of essential importance. Furthermore, the leadership style of the ward manager in the practicum placement [24, 25, 39] and students’ self-reported theoretical knowledge and practical skills at the beginning of the clinical practicum [8] are seen to be associated with a positive clinical practicum experience.

The GNSs represent a critical part of the nursing workforce in solving the nursing shortage in Europe [40]. It is evident that good experiences of the final clinical practicum enhance a successful transition and socialization toward working life, which may have a long-lasting effect on professional commitment and avoiding nurses’ intentions to leave the profession [2, 10, 41]. In this light, it becomes evident that students require a student-centric [3, 24], positive and supportive CLE [3]. In general, European nursing students have reported clinical practicum as being a positive experience [8, 24]. Despite this, there are several studies reporting variations in students’ clinical practicum experiences between countries, especially NT cooperation and factors associated with it, which remain underexposed in the literature [8, 17, 24, 36]. Therefore, the aim of this study was to explore and compare GNSs’ experiences of NT cooperation, the CLE and supervision in their final clinical practicum, and to analyze factors associated with these experiences in six European countries.

The following research questions were addressed:

1. What kind of experiences do GNSs have of NT cooperation?
2. What kind of experiences do GNSs have of the CLE and supervision?
3. What factors are associated with the GNSs’ experiences of NT cooperation, and the CLE and supervision?

## Methods

### Design

The study used a cross-sectional international comparative survey design and is a part of the European Professional Competence of Nursing (PROCOMPNurse) -project aiming to assess the level of competence of GNSs at the transition period in six European countries. The study describes the clinical role of the NT, whereas the academic role of the NT is already published from this data elsewhere [42]. STROBE cross-sectional reporting guidelines were used in this study [43].

### Sample and setting

A convenience sample of GNSs (*N* = 3675, *n* = 1796) from 45 educational institutions in Finland, Germany, Iceland, Ireland, Lithuania and Spain was recruited. These countries were included because their nursing studies are provided either in higher education institutions (HEIs i.e., universities and university of applied sciences) or in nursing colleges or nursing schools [35]. The length of the nursing degree programmes varies from 3 to 4 years and from 210 to 240 European Credit Transfer and Accumulation System (ECTS) credits between the participating countries. Nursing students were eligible for the study if they (1) studied in a nursing degree programme based on European Union directives [5, 33] leading to a qualification as an RN, (2) took their final clinical practicum at the stage of graduation, and (3) conducted the clinical practicum in adult patient units.

The sample sizes, as determined by power analysis, showed that 156 nursing students per country were needed [44]. The number of the total GNS population per year is different in each partner country, thus the sample sizes varied nationally. National geographical representativeness was considered when applicable.

### Data collection

The data were collected between May 2018 and March 2019. The online survey was performed with REDCap (https://www.project-redcap.org/) electronic software via a shared link during a class or via email and with a reminder email 2 weeks later. A paper-based version with data collection during a class was used in cases where the online version was not possible to use. The national research team and the contact persons for each educational institution were responsible for the identification and recruitment of potential student participants, distributing the questionnaires in their organization and ensuring that it was available to all GNSs meeting the inclusion criteria.

A modified version of the Clinical Learning Environment and Supervision and Nurse Teacher (hereafter CLES+T) Scale [16] was used for the first time in this study to assess GNSs’ experience of the NT cooperation, the CLE and supervision in their final clinical practicum. The original CLES+T Scale [16] was modified together with the copyright holder of the CLES+T Scale to make it suitable for this study. This modification was made to explore the diverse and changed clinical role of NTs in the European arena in more detail. For this purpose, the original T -subscale with 9 items was replaced with a new subscale, NT’s pedagogical cooperation with students (hereafter T -cooperation), with 5 items assessing: Individual supervision, Relieving stress, Promoting learning, NT’s response time and Ease of cooperation [7]. GNSs’ experiences of the CLE and supervision were examined in this study with the following original 4 subscales (25 items) forming the CLES Scale: Pedagogical atmosphere (9 items), Leadership style of the ward manager (4 items), Premises of the care of ward (4 items) and Content of the supervisory relationship (8 items). A five-point Likert scale from 1 (fully disagree) to 5 (fully agree) was used. The internal consistency measured with the Cronbach’s alpha was 0.96 for the CLES Scale and 0.88 for the T -cooperation subscale and ranged from 0.83 to 0.85 for the items of this subscale. Moreover, the internal consistency of the T -subscale ranged between countries from 0.85 to 0.91 (Table 2).

The structured questionnaire consisted of two parts. The first part consisted of individual background factors (gender, age, nursing as a first study option, work experience in healthcare, current nursing program) and educational background factors (satisfaction with theoretical studies in their current program, satisfaction with clinical practicum, NT’s involvement in supervision, main form of the supervisory relationship) shown to be associated with the nursing students’ positive experience of the clinical practicum [17]. The second part consisted of the modified Clinical Learning Environment and Supervision (CLES+T) Scale (Table 1). As there were no previous translations, the T -subscale was translated into national languages (German, Icelandic, Irish, Lithuanian, Spanish) and, in addition, the CLES Scale was translated into Icelandic using the backtranslation method [45]. The national questionnaires were piloted before data collection to ensure their comprehensibility and clarity.

**Table 1:** Background factors and significant associations with GNSs’ experiences of NT cooperation, the CLE and supervision

	Total *N* = 1796	Background factors							T -cooperation subscale Total mean			CLES Scale Total mean		
		Finland *n* = 514	Germany *n* = 304	Iceland *n* = 64	Ireland *n* = 399	Lithuania *n* = 272	Spain *n* = 240	*p-value* ^a ^	Model based mean estimate	95% CI	*p-value* ^b^	Model based mean estimate	95% CI	*p-value* ^b^
**Individual factors**														
**Nursing as a first study option**	n (%)	n (%)	n (%)	n (%)	n (%)	n (%)	n (%)	< 0.0001			0.51			0.052
Yes	1262 (70.9)	447 (88.3)	177 (58.8)	40 (62.5)	280 (70.5)	163 (60.2)	155 (64.6)							
No	517 (29.0)	59 (11.7)	124 (41.2)	24 (37.5)	117 (29.5)	108 (39.9)	85 (35.4)							
**Current nursing program**								< 0.0001			0.30			0.58
HEI degree (university or university of applied sciences)	1254 (73.5)	504 (100.0)		63 (100.0)	361 (99.5)	87 (32.3)	239 (100.0)							
College or nursing school degree	453 (26.5)		269 (100.0)		2 (0.6)	182 (67.7)								
**Educational factors**														
**Satisfaction with theoretical studies**								< 0.0001			< 0.0001			< 0.0001
Very satisfied	430 (27.0)	21 (4.4)	20 (6.9)	8 (16.7)	39 (11.0)	52 (20.2)	33 (19.8)		4.4	4.2-4.5		3.9	3.8-4.0	
Satisfied	889 (55.9)	223 (46.7)	144 (49.3)	37 (77.1)	212 (59.7)	164 (63.6)	102 (61.1)		4.0	3.9-4.0		3.8	3.7-3.9	
Unsatisfied	225 (14.2)	202 (42.3)	106 (36.3)	3 (6.3)	91 (25.6)	36 (14.0)	28 (16.8)		3.5	3.4-3.7		3.7	3.6-3.8	
Very unsatisfied	46 (2.9)	32 (6.7)	22 (7.5)	0 (0.0)	13 (3.7)	6 (2.3)	4 (2.4)		3.2	2.9-3.5		3.6	3.3-3.6	
**Satisfaction with clinical practicum**								< 0.0001			< 0.0001			< 0.0001
Very satisfied	430 (27.0)	155 (32.6)	45 (15.4)	12 (25.5)	86 (24.4)	79 (30.9)	53 (31.7)		4.2	4.1-4.3		4.2	4.1-4.3	
Satisfied	889 (55.9)	274 (57.6)	160 (54.8)	33 (70.2)	197 (56.0)	136 (53.1)	89 (53.3)		3.9	3.8-4.0		3.8	3.8-3.9	
Unsatisfied	225 (14.2)	39 (8.2)	72 (24.7)	2 (4.3)	59 (16.8)	33 (12.9)	20 (12.0)		3.6	3.4-3.8		3.4	3.3-3.5	
Very unsatisfied	46 (2.9)	8 (1.7)	15 (5.1)	0 (0.0)	10 (2.8)	8 (3.1)	5 (3.0)		3.5	3.1-3.9		3.4	3.2-3.6	
**NT cooperation with student**								< 0.0001						< 0.0001
Yes	826 (53.2)	269 (56.4)	88 (32.5)	36 (75.0)	101 (30.0)	187 (73.6)	145 (87.9)					3.8	3.7-3.9	
No	726 (46.8)	208 (43.6)	183 (67.5)	12 (25.0)	236 (70.0)	67 (26.4)	20 (12.1)					3.6	3.5-3.7	
**Main form of the supervisory relationship**								< 0.0001			0.004			< 0.0001
Named individual mentor	736 (54.8)	352 (78.4)	129 (50.2)	11 (25.0)	94 (49.2)	64 (26.8)	86 (52.8)		4.0	3.9-4.1		3.8	3.7-4.0	
Group supervision	111 (8.3)	18 (4.0)	8 (3.1)	4 (9.1)	4 (2.1)	41 (17.2)	36 (22.1)		3.7	3.5-3.9		3.7	3.5-3.8	
Changing supervision	341 (25.4)	69 (15.4)	116 (45.1)	28 (63.6)	44 (23.0)	56 (23.4)	28 (17.2)		3.7	3.6-3.9		3.6	3.5-3.7	
Named clinical placement coordinator or equivalent	155 (11.5)	10 (2.2)	4 (1.6)	1 (2.3)	49 (25.7)	78 (32.6)	13 (8.0)		4.0	3.8-4.2		3.7	3.6-3.9	

*GNSs* Graduation nursing students, *NT* Nurse teacher, *CLE* Clinical learning environment, *T -cooperation* Nurse teacher’s pedagogical cooperation with students, *CLES* Clinical Learning Environment and Supervision, *CI* Confidence interval, *HEI* Higher education institution

^a^ *p*-values are calculated using a chi-square test

^b^ *p*-values are calculated using with the linear model

### Analyses

To present the data, frequencies (n) and percentages, or mean and standard deviation (SD), were calculated depending on the nature of the variable. A Chi-Square test was conducted to analyze differences between countries both in the categorized total mean T -cooperation scores and in the categorized total mean CLES scores. The association between the total mean T -cooperation score and the total mean CLES score and individual background factors (country, nursing as first study option, work experience in healthcare, current nursing program) and educational background factors (satisfaction with theoretical studies, satisfaction with clinical practicum, meeting with NT, main form of the supervisory relationship) were analyzed with the linear model, including the country and one individual or educational background factor. Only significant factors were left in the final model. Assumptions of normal distributions were checked from studentized residuals (e.g. using a normal quantile plot). A Kruskal-Wallis test was used to compare item levels of the T -cooperation subscale between the countries while assumptions for the parametric test were not met. After the overall test, pairwise comparisons were adjusted using the Stell-Dwass method. Mean values are presented despite nonparametric testing to be able to present the differences between the countries. Dependencies between the T -cooperation subscale and CLES Scale were examined with Pearson’s correlation coefficient.

Model-based means were estimated to describe the association between the T -cooperation subscale or CLES Scale and the background variable. Cronbach’s alpha was used to assess the internal consistency of the scales used in this study. The statistical significance level was set at values of *p* < 0.05 (two-tailed). Moreover, 95% confidence intervals (CIs) were calculated. All analyses were performed using SAS version 9.4 for Windows software (SAS Institute Inc., Cary, NC, USA).

### Ethics

Ethical principles [46–48] were complied with throughout the study. The study was approved by the Ethics Committee of the University of Turku (Statement 62/2017, 11.12.2017) and national ethical approvals were received when needed. Permission to conduct the study was obtained from the local educational institution according to national and organizational standards. Permissions to use and translate the original CLES Scale and the T -cooperation subscale were received from the copyright holders. The study was conducted on a voluntary basis and the GNSs were informed orally and in writing about the study and their right to withdraw at any time. Participating GNSs gave their written informed consent before completing the survey.

## Results

### Participants

Out of 3676 recruited GNSs, a total of 1796 GNSs (total response rate = 49%, range 36–88%) from 45 educational institutions in six countries responded to the questionnaire (Table 1). The GNSs were predominantly female (*n* = 1563, 88.0%) and the median age was 23, ranging from 18 to 60 years. The majority (*n* = 1254, 73.5%) were studying at an HEI and the rest either at a university college in Lithuania or at a nursing school in Germany. More than half of the GNSs (*n* = 1079, 60.7%) reported prior work experience in healthcare, ranging from 0 to 360 months. Half of the GNSs (*n* = 826, 53.2%) had an NT involved in their supervision and more than half had a named individual mentor in their last clinical practicum (*n* = 736, 54.8%). Significant differences were found between countries regarding all background factors, both individual and educational (Table 1).

### Students’ experiences of NT cooperation

Overall, the GNSs had positive experiences of NT cooperation (total mean 3.9, SD 0.9, range 1.0–5.0). GNSs in Spain showed the highest subscale mean score of 4.3 (SD 0.8), while GNSs in Ireland had the lowest subscale mean score of 3.7 (SD 1.0). At the item level, mean scores varied between 3.5 and 4.4, with the highest means in items assessing the individual supervision of NTs (mean 4.2, SD 0.9) and relieving stress (mean 4.1, SD 1.0). GNSs in Spain reported the highest item mean scores, ranging from 3.9 to 4.4 in all items, while GNSs in Ireland reported the lowest, ranging from 3.1 to 4.0 (Table 2).

**Table 2:** GNSs’ final clinical practicum experiences of the NT cooperation, and the CLE and supervision

	Total n Mean (SD) range	Finland n Mean(SD) range	Germany n Mean (SD) range	Iceland n Mean (SD) range	Ireland n Mean (SD) range	Lithuania n Mean (SD) range	Spain n Mean (SD) range	*p-value*	*Cronbach’s α*
**The nurse teacher’s pedagogical cooperation with students**	823 3.9 (0.9) 1.0-5.0	269 3.8 (0.9) 1.0-5.0	88 3.8 (0.9) 1.6-5.0	36 4.0 (0.6) 2.8-5.0	99 3.7 (1.0) 1.0-5.0	187 4.0 (0.8) 1.6-5.0	144 4.3 (0.8) 1.4-5.0	< 0.0001^a^	0.88
*Individual supervision*	823 4.2 (0.9) 1.0-5.0	269 4.3 (0.9) 1.0-5.0	88 4.1 (0.9) 2.0-5.0	36 4.2 (0.6) 3.0-5.0	99 4.0 (1.2) 1.0-5.0	85 4.3 (0.8) 2.0-5.0	144 4.4 (0.9) 1.0-5.0	< 0.0001^b^	0.85
*Relieving stress*	821 4.1 (1.0) 1.0-5.0	269 4.1 (1.0) 1.0-5.0	88 3.9 (0.9) 2.0-5.0	36 4.2 (0.6) 3.0-5.0	99 3.9 (1.2) 1.0-5.0	86 4.2 (0.9) 2.0-5.0	143 4.4 (1.0) 1.0-5.0	< 0.0001^b^	0.85
*Promoting learning*	822 4.0 (1.0) 1.0-5.0	269 3.8 (1.0) 1.0-5.0	88 3.9 (1.1) 1.0-5.0	36 4.1 (0.8) 2.0-5.0	99 3.8 (1.1) 1.0-5.0	85 4.0 (1.0) 1.0-5.0	143 4.3 (0.9) 1.0-5.0	< 0.0001^b^	0.83
*Teacher’s response time*	821 3.5 (1.2) 1.0-5.0	269 3.2 (1.3) 1.0-5.0	87 3.3 (1.1) 1.0-5.0	36 3.5 (1.2) 1.0-5.0	99 3.5 (1.2) 1.0-5.0	85 3.8 (1.2) 1.0-5.0	143 3.9 (1.1) 1.0-5.0	< 0.0001^b^	0.85
*Ease of cooperation*	819 3.7(1.2) 1.0-5.0	269 3.5 (1.2) 1.0-5.0	88 3.7 (1.2) 1.0-5.0	36 3.9 (1.3) 2.0-5.0	99 3.1 (1.3) 1.0-5.0	87 3.8 (1.2) 1.0-5.0	143 4.3 (0.9) 1.0-5.0	< 0.0001^b^	0.87
*Cronbach’s α*	0.86	0.86	0.88	0.85	0.91	0.87	0.89		
**The learning environment and the content of the supervisory relationship**									
Pedagogical atmosphere	1607 4.0 (0.8) 1.0-5.0	480 4.1 (0.8) 1.0-5.0	299 3.7 (0.9) 1.0-5.0	48 3.9 (0.6) 2.3-5.0	358 3.9 (0.8) 1.2-5.0	259 4.0 (0.8) 1.0-5.0	163 4.2 (0.8) 1.4-5.0	< 0.0001^a^	0.84
Leadership style of the ward manager (WM)	1603 3.9 (0.9) 1.0-5.0	479 3.8 (0.9) 1.0-5.0	299 3.6 (1.0) 1.0-5.0	47 4.0 (0.9) 2.0-5.0	357 4.0 (1.0) 1.0-5.0	258 4.0 (0.8) 1.0-5.0	163 3.9 (1.0) 1.0-5.0	< 0.0001^a^	0.85
Nursing care on the ward	1603 3.9 (0.8) 1.0-5.0	478 3.9 (0.8) 1.0-5.0	300 3.4 (0.9) 1.0-5.0	47 4.0 (0.7) 2.3-5.0	357 3.9 (0.8) 1.3-5.0	258 4.1 (0.8) 1.5-5.0	163 4.2 (0.7) 2.0-5.0	< 0.0001^a^	0.85
The content of the supervisory relationship	1598 4.0 (0.9) 1.0-5.0	478 4.2 (0.9) 1.0-5.0	297 3.8 (1.1) 1.0-5.0	47 4.2 (0.6) 2.4-5.0	356 3.9 (0.9) 1.0-5.0	257 4.1 (0.8) 1.0-5.0	163 4.3 (0.9) 1.0-5.0	< 0.0001^a^	0.84
Total mean^c^	1648 4.0 (0.7) 1.1-5.0	514 4.1 (0.7) 1.1-5.0	302 3.7 (0.8) 1.2-5.0	48 4.0 (0.6) 2.9-5.0	362 3.9 (0.7) 1.1-5.0	259 4.1 (0.7) 1.1-5.0	163 4.2 (0.7) 1.8-5.0	< 0.0001^a^	0.96

A five-point Likert scale from 1 (fully disagree) to 5 (fully agree) was used

*GNSs* Graduation nursing students, *NT* Nurse teacher, *CLE* Clinical learning environment, *SD* Standard deviation

^a^ *p*-values are calculated between countries using a Chi-square test

^b^*p*-values are calculated between countries using a Kruskall-Wallis test

^c^Total mean consists of CLES+T scale, where the original T -subscale is replaced with a T -cooperation subscale

Between the countries, there were statistically significant differences both on the subscale level (*p* < 0.001) and item levels of the T -cooperation subscale (*p* < 0.004). On the subscale level, the highest statistically significant between-country difference was detected between GNSs in Spain and Ireland with a difference of 0.6 (CI 95% from 0.8 to 0.4, *p* < 0.001), while the lowest was between Germany and Lithuania with a difference of 0.2 (CI 95% from 0.0 to 0.4, *p* = 0.04) (Table 2).

### Students’ experiences of the CLE and supervision

Overall, the GNSs had positive experiences of the CLE and supervision (total mean 4.0, SD 0.74, range 1.1–5.0). Between the countries, there were statistically significant differences both in the total mean CLES (*p* < 0.001) and at all subscales (*p* < 0.0001). GNSs in Spain showed the highest total mean CLES score of 4.2 (SD 0.7), while GNSs in Germany had the lowest total mean score of 3.7 (SD 0.8) (Table 2). The highest statistically significant between-country difference was detected between GNSs in Spain and Germany with a difference of 0.5 (CI 95% from 0.7 to 0.4, *p* < 0.001). The lowest statistically significant between-country difference was detected between Finland and Spain with a difference of 0.1 (CI 95% from 0.3 to 0.01, *p* = 0.03).

At the subscale level of the CLES, total means varied between 3.9 and 4.0, with the highest means in subscales Pedagogical atmosphere (mean 4.0, SD 0.8) and Supervisory relationship (mean 4.0, SD 0.9). GNSs in Spain reported the highest total mean scores, ranging from 4.2 to 4.3 in all subscales expect in the subscale Leadership style of the ward manager. GNSs in Germany reported the lowest total mean scores, ranging from 3.4 to 3.7 in all subscales (Table 2).

### Factors associated with NT cooperation, the CLE and supervision

Statistically significant associations were detected between students’ background factors and students’ experiences of NT cooperation, the CLE and supervision. GNSs in Spain (*p* < 0.0001), GNSs with a named individual mentor (*p* < 0.0001), and those who were very satisfied with the theoretical studies (*p* = 0.02), reported higher total mean scores of the T-cooperation subscale than others. GNSs in Spain, GNSs with a named individual mentor, nurse teacher involvement in student supervision, and those who were very satisfied with the theoretical studies, reported higher total mean CLES scores (all *p* < 0.001) than others.

### The association between the T -cooperation subscale and the CLES scale

In the total sample, a statistically significant moderate positive correlation was detected between the T -cooperation subscale and the CLES Scale (*r* = 0.40, *p* < 0.0001). A statistically significant moderate positive correlation was detected between all five items of the T -cooperation subscale and all four subscales of the CLES Scale. Pearson Correlation Coefficients ranged from 0.19 to 0.36 (all *p* < 0.0001) indicating a weak to moderate positive linear correlation between the T -cooperation subscale and the CLES Scale (Table 3).

**Table 3:** Pearson’s correlation coefficients between the T -cooperation subscale and the CLES Scale

Subscales of the CLES Scale	NT’s pedagogical cooperation with students	
	*R* ^a^	*p-value*
Pedagogical atmosphere	0.33	< 0.0001
Leadership style of the ward manager	0.28	< 0.0001
Premises of the care on the ward	0.28	< 0.0001
The content of the supervisory relationship	0.39	< 0.0001
Total CLES	0.40	< 0.0001

*T -cooperation* NT’s pedagogical cooperation with students, *CLES* Clinical Learning Environment and Supervision, *NT* Nurse teacher

^a^The Pearson’s correlation coefficient was interpreted as follows: 0.10 to 0.39 weak, 0.40 to 0.69 moderate, 0.70 to 0.96 strong

In all countries, a statistically significant positive correlation was found between the T -cooperation subscale and the CLES Scale. Pearson Correlation Coefficients ranged from 0.28 to 0.70 (*p* values ranged from 0.0023 to < 0.0001), indicating a weak to strong positive linear correlation between NT cooperation, the CLE and supervision in the final clinical practicum of GNSs.

## Discussion

The overall results of this study are promising for European nursing education and clinical practice. In all participating countries, the GNSs’ experiences of NT cooperation, the CLE and supervision were rated highly, indicating positive experiences. This is in line with earlier European studies with students in different stages of their studies [8, 24, 36]. The final clinical practicum of GNSs should provide them with positive experiences and opportunities to face both the reality of nursing practice and the role of the nurse [2]. Such a strategy encourages GNSs to become clinically confident and competent and has the potential to boost the transition of students to the role of the professional nurse [1, 9] and to remain in their newly graduated profession [2].

This study deepened the knowledge base of European NT cooperation, which has been rarely investigated [7]. As the results show, NT cooperation varies both between European countries and on the national level. NT cooperation appeared to influence the GNSs’ final clinical practicum experiences positively; those GNSs who had an NT cooperating with them reported more positive experiences than GNSs without this cooperation. This finding is supported by earlier studies [14, 23] and may indicate the importance of the student-centric [3, 24] and supportive CLE [3]. However, NT cooperation has been a priority, especially in Iceland and Spain, where the NT has been assigned with a clear clinical role: In Iceland, a nurse NT (called Clinical Instructors, CIs) consistently meets students on a weekly basis during the clinical practicum, i.e. provides feedback on clinical work, engages in clinical reasoning, encourages independence, and fosters critical thinking based on evidence [49]. In Spain, NTs (called Academic Mentors) act as a “bridge” between the university and the clinical institution, helping students to integrate concepts and guide their reflection during mentoring sessions. Academic Mentors meet both students and nurses in clinical settings and take part in the students’ learning and assessing process. Conversely, Ireland is in a unique position with both a Clinical Placement Coordinator (CPC) and an NT involved in the supervision of nursing students, albeit the NT is without any clear clinical role. According to the Nursing and Midwifery Board of Ireland, [50] the CPC is a registered nurse who promotes the CLE by supporting, facilitating and monitoring the clinical learning of students. The CPC’s role is highly valued as a form of Practice-Based Teachers who support both the mentors and students in clinical practice. Moreover, the clinical role of the NT is diverse in Ireland; some HEIs have a clear NT role while other HEI teachers attend the clinical placements when there is a need to offer support in relation to the assessment of a clinical practicum or when a student fails a clinical practicum.

Significant differences in GNSs’ experiences of NT cooperation in the final clinical practicum were identified between the countries. In Spain, GNSs had the most positive experiences, both of NT cooperation and the CLE and supervision. This may be because the image of nurses in Spanish society has improved in recent years [51], and therefore a larger number of students enter nursing in Spain. In addition, there are good employment opportunities after graduation, and there are vacancies in various clinical settings, such as private and public health centers, hospitals, and nursing homes. However, this may be partly explained with the legal document [52] regulating the rights and duties of students, mentors, and NTs during the clinical practicum in Spain, thus providing more structure for the supervision of nursing students. The presence of an NT who meets students during clinical practicum maintains a connection between academic and clinical learning and helps students integrate practical and theoretical content. It is evident that these country differences need further research to ensure that European nursing education has a uniform implementation to enhance the quality and adequacy of the future nursing workforce [2, 40].

The results are in line with earlier studies indicating that GNSs highly value the individual supervision from their NT [14, 22], even though it is often reported by students to be unattainable [14, 18]. Moreover, this study reveals that GNSs value the individual supervision of the NT as well as support of the NT for promoting learning and relieving clinical practicum-related stress, even when there are challenges in the NT’s response time to the GNS’s requests for cooperation and in the ease of cooperation. It appears that GNSs expectations of their NT do not fully reflect the actual implementation of the NT cooperation. Thus, new evidence-based cooperation methods with novel alternatives need to be developed. In future, rigorous research is needed to evaluate the usability and utility as well as effectiveness of digital technologies in NT cooperation to enhance the high-quality clinical practicum of nursing students. As a result, the NT’s competence in cooperation and technology literacy should also be assessed to enable a full potential of digital technologies in NT cooperation. There is also a need to update the European Union directives to more clearly define the clinical role of NTs in Europe to enable them to have the main responsibility for student supervision in clinical practicum.

Significant differences in GNSs’ experiences of CLE and supervision were identified between the countries. These differences may be due to national disparities in the implementations [36] between educational institutions and between the countries involved. It is noteworthy that Ireland was the only country where the length of the last clinical practicum was constant, set to a long period of 36-weeks by the Nursing and Midwifery Board of Ireland’s (NMBI) Standards and Requirements [50].

The subscales Supervisory relationship and Pedagogical atmosphere on the ward were rated highest among GNSs, as is also seen in several earlier studies [6, 22, 39, 41]. Thus, the finding highlights the importance of the mentor supervisory relationship [8, 24] and the pedagogical atmosphere in the ward [8]. However, GNSs in Germany studying at a diploma level at nursing school generally rated their experiences poorly when compared to students in other countries studying at HEIs. These experiences of GNSs in Germany may be due to the common shortage of staff in the practicum settings [53], leading to a situation where students are scheduled as full workers on the practicum ward and with an overworked mentor having limited resources to supervise the student. Moreover, there is a lack of both official guidelines and mentor training of clinical practicum in Germany which may impact the quality of the CLE [54–56].

In this study, GNSs’ individual background factors did not affect the students’ experiences, albeit some educational background factors were shown to be significantly associated with GNSs’ experiences of NT cooperation, the CLE and supervision. First, this study reveals the importance of the NT’s role in the final clinical practicum, which is not supported with earlier evidence [8]. Second, the student satisfaction with their learning processes during the degree studies seem to be important in positively affecting GNSs’ final clinical practicum experiences. Those satisfied with theoretical studies reported more positive experiences of both NT cooperation, the CLE and supervision, confirming earlier evidence [7]. Moreover, GNSs with a named individual mentor reported more positive experiences of the CLE and supervision, which gives a reason to continue an individualized, one-to-one supervisory relationship in clinical practicums in Europe [6, 33]. However, it should be noted that there is also a growing shortage of clinical practicum placements in Europe, and the supervision of students is increasingly organized by models of supervision in student-dedicated units [26, 57, 58], albeit this model requires further research.

### Strengths and limitations

The individual country subsamples collected in this study may not be representative of the national GNS population. However, the electronic survey that was enabled to conduct data collection in several European countries, and the sample of this study, represents a European cohort gathered from several educational institutions in six countries. The total response rate was moderate for the electronic survey, less than half (49%) of the total sample, which is better than the common low response rate associated with electronic surveys [59]. On the European level, the sample may be considered representative; student gender and age distribution were rather similar to European nursing students in general [19, 36]. GNSs in Spain had the lowest response rate at 36%, which should be taken into account when considering the findings of this study. It is possible that those Spanish GNSs who responded to the survey had the most positive experiences or might have answered in a socially desirable manner. Those who had worse experiences might not have answered the survey. However, this was not possible to control in authentic learning environments; GNSs’ clinical placement settings and the pedagogical solutions used in theoretical and clinical studies were not included in the data collection protocol. In the future, these aspects are recommended to be included in international comparative studies in order to be able to interpret country variations in the above-mentioned aspects more deeply.

## Conclusion

Overall, NT cooperation, the CLE and supervision were rated at a good level in all participating countries. This study reveals the importance of the clinical role of NTs in supporting and guiding the GNSs’ final clinical practicum, but the cooperation is not always optimal. Therefore, more attention should be paid to the unique NT cooperation when designing the clinical practicum implementations, methods of cooperation and NT resources. Moreover, an emphasis on seamless collaboration between educational institutions and healthcare organizations is needed in establishing NT cooperation that is beneficial for the success of nursing students in their clinical practicum, which thus may alleviate the existing nursing shortage. In the future, international longitudinal comparative research throughout all nursing degree studies to the working life of the student is required. In addition, it should be examined whether student learning outcomes and quality of care are associated with positive student experiences of NT cooperation, the CLE and supervision.

## Data Availability

The datasets used and/or analyzed during the current study are available from the corresponding author on reasonable request.

## Abbreviations

CI: Confidence interval
CLE: Clinical Learning Environment
CLES Scale: Clinical Learning Environment and Supervision Scale
CLES+T Scale: Clinical Learning Environment, Supervision and Nurse Teacher Scale
GNS: Graduating nursing student
HEI: Higher education institution
NT: Nurse teacher
SD: Standard deviation

## References

[1] Järvinen T, Eklöf N, Salminen L (2018). Factors related to nursing students’ readiness to enter working life - a scoping literature review. Nurse Educ Pract.

[2] Kaihlanen AM, Elovainio M, Haavisto E, Salminen L, Sinervo T (2020). Final clinical practicum, transition experience and turnover intentions among newly graduated nurses: a cross sectional study. Nurse Educ Today.

[3] Phillips KF, Mathew L, Aktan N, Catano B (2017). Clinical education and student satisfaction: an integrative literature review. Int J Nurs Sci.

[4] Kaihlanen AM, Gluschkoff K, Koskinen S, Salminen L, Strandell-Laine C, Fuster Linares P, et al. Final clinical practicum shapes the transition experience and occupational commitment of newly graduated nurses in Europe-a longitudinal study. J Adv Nurs. 2021. 10.1111/jan.15060. 10.1111/jan.15060 PMC9293159 34626003

[5] Directive 2005/36/EC of the European parliament and of the council of 7 September 2005 on the recognition of professional qualifications. European Commission; 2005. http://eur-lex.europa.eu/legal-content/EN/TXT/PDF/?uri=CELEX:32005L0036&rid=1. Cited 2021 Oct 23.

[6] Warne T, Johansson UB, Papastavrou E, Tichelaar E, Tomietto M, Van den Bossche K, Moreno MF, Saarikoski M (2010). An exploration of the clinical learning experience of nursing students in nine European countries. Nurse Educ Today.

[7] Strandell-Laine C, Saarikoski M, Löyttyniemi E, Meretoja R, Salminen L, Leino-Kilpi H (2018). Effectiveness of mobile cooperation intervention on students’ clinical learning outcomes: a randomized controlled trial. J Adv Nurs.

[8] Cant R, Ryan C, Cooper S (2021). Nursing students’ evaluation of clinical practice placements using the clinical learning environment, supervision and nurse teacher scale - a systematic review. Nurse Educ Today.

[9] Kaihlanen AM, Salminen L, Flinkman M, Haavisto E (2019). Newly graduated nurses’ perceptions of a final clinical practicum facilitating transition: a qualitative descriptive study. Collegian.

[10] van Rooyen DRM, Jordan PJ, Ten Ham-Baloyi W, Caka EM (2018). A comprehensive literature review of guidelines facilitating transition of newly graduated nurses to professional nurses. Nurse Educ Pract.

[11] Cleary M, Visentin D, West S, Lopez V, Kornhaber R (2018). Promoting emotional intelligence and resilience in undergraduate nursing students: an integrative review. Nurse Educ Today.

[12] Collard SS, Scammell J, Tee S (2020). Closing the gap on nurse retention: a scoping review of implications for undergraduate education. Nurse Educ Today.

[13] Bakker EJM, Kox JHAM, Miedema HS, Bierma-Zeinstra S, Runhaar J, Boot CRL, van der Beek AJ, Roelofs PDDM (2018). Physical and mental determinants of dropout and retention among nursing students: protocol of the SPRiNG cohort study. BMC Nurs.

[14] Heinonen AT, Kääriäinen M, Juntunen J, Mikkonen K (2019). Nursing students’ experiences of nurse teacher mentoring and beneficial digital technologies in a clinical practice setting. Nurse Educ Pract.

[15] Järvinen T, Virtanen H, Kajander-Unkuri S, Salminen L (2021). Nurse educators’ perceptions of factors related to the competence of graduating nursing students. Nurse Educ Today.

[16] Saarikoski M, Isoaho H, Warne T, Leino-Kilpi H (2008). The nurse teacher in clinical practice: developing the new sub-dimension to the clinical learning environment and supervision (CLES) scale. Int J Nurs Stud.

[17] Strandell-Laine C, Leino-Kilpi H, Löyttyniemi E, Salminen L, Stolt M, Suomi R, Saarikoski M (2019). A process evaluation of a mobile cooperation intervention: a mixed methods study. Nurse Educ Today.

[18] Gidman J, McIntosh A, Melling K, Smith D (2011). Student perceptions of support in practice. Nurse Educ Pract.

[19] Saarikoski M, Kaila P, Lambrinou E, Pérez Cañaveras RM, Tichelaar E, Tomietto M, Warne T (2013). Students’ experiences of cooperation with nurse teacher during their clinical placements: an empirical study in a Western European context. Nurse Educ Pract.

[20] Skaalvik AW, Henriksen N, Normann HK (2015). The nurse teacher’s role in clinical practice - Norwegian nursing students’ experiences. A cross-sectional survey. Nordisk sygeplejeforskning.

[21] Gustafsson M, Kullén Engström A, Ohlsson U, Sundler AJ, Bisholt B (2015). Nurse teacher models in clinical education from the perspective of student nurses-a mixed method study. Nurse Educ Today.

[22] Papastavrou E, Dimitriadou M, Tsangari H, Andreou C (2016). Nursing students’ satisfaction of the clinical learning environment: a research study. BMC Nurs.

[23] Suikkala A, Timonen L, Leino-Kilpi H, Katajisto J, Strandell-Laine C (2021). Healthcare student-patient relationship and the quality of the clinical learning environment - a cross-sectional study. BMC Med Educ.

[24] Pitkänen S, Kääriäinen M, Oikarainen A, Tuomikoski A, Elo S, Ruotsalainen H (2018). Healthcare students’ evaluation of the clinical learning environment and supervision - a cross-sectional study. Nurse Educ Today.

[25] Adam AB, Druye AA, Kumi-Kyereme A, Osman W, Alhassan A (2021). Nursing and midwifery students’ satisfaction with their clinical rotation experience: the role of the clinical learning environment. Nurs Res Pract.

[26] Manninen K, Henriksson EW, Scheja M, Silén C (2015). Supervisors’ pedagogical role at a clinical education ward - an ethnographic study. BMC Nurs.

[27] Gusar I, Bačkov K, Tokić A, Dželalija B, Lovrić R. Nursing student evaluations on the quality of mentoring support in individual, dual and group approaches during clinical training: a prospective cohort study. Aust J Adv Nurs. 2020;37(4). 10.37464/2020.374.83.

[28] Al-Anazi NA, Alosaimi D, Pandaan I, Anthony D, Dyson S (2019). Evaluating clinical placements in Saudi Arabia with the CLES+T scale. Nurse Educ Pract.

[29] Kukkonen P, Suhonen R, Salminen L (2016). Discontinued students in nursing education - who and why?. Nurse Educ Pract.

[30] Carlson E, Idvall E (2014). Nursing students’ experiences of the clinical learning environment in nursing homes: a questionnaire study using the CLES+T evaluation scale. Nurse Educ Today.

[31] Roso-Bas F, Pades Jiménez A, García-Buades E (2016). Emotional variables, dropout and academic performance in Spanish nursing students. Nurse Educ Today.

[32] Davies R (2008). The Bologna process: the quiet revolution in nursing higher education. Nurse Educ Today.

[33] Directive 2013/55/EC of 20 November 2013 on the recognition of professional qualifications. Amendment to directive 2005/36/EC of the European Parliament and of the Council. European Commission; 2005. http://eur-lex.europa.eu/legal-content/EN/TXT/PDF/?uri=CELEX:32013L0055&rid=1. Cited 2021 Oct 23.

[34] Antohe I, Riklikiene O, Tichelaar E, Saarikoski M (2016). Clinical education and training of student nurses in four moderately new European Union countries: assessment of students’ satisfaction with the learning environment. Nurse Educ Pract.

[35] Lahtinen P, Leino-Kilpi H, Salminen L (2014). Nursing education in the European higher education area - variations in implementation. Nurse Educ Today.

[36] Visiers-Jiménez L, Suikkala A, Salminen L, Leino-Kilpi H, Löyttyniemi E, Henriques MA, Jiménez-Herrera M, Nemcová J, Pedrotti D, Rua M, Tommasini C, Zeleníková R, Kajander-Unkuri S (2021). Clinical learning environment and graduating nursing students’ competence: a multi-country cross-sectional study. Nurs Health Sci.

[37] Kukkonen P, Leino-Kilpi H, Koskinen S, Salminen L, Strandell-Laine C (2020). Nurse managers’ perceptions of the competence of newly graduated nurses: a scoping review. J Nurs Manag.

[38] González-García A, Díez-Fernández A, Leino-Kilpi H, Martínez-Vizcaíno V, Strandell-Laine C (2021). The relationship between clinical placement duration and students’ satisfaction with the quality of supervision and learning environment: a mediation analysis. Nurs Health Sci.

[39] Bisholt B, Ohlsson U, Engström AK, Johansson AS, Gustafsson M (2014). Nursing students’ assessment of the learning environment in different clinical settings. Nurse Educ Pract.

[40] World Health Organization (2020). Health workforce, data and statistics.

[41] Rodríguez-García MC, Gutiérrez-Puertas L, Granados-Gámez G, Aguilera-Manrique G, Márquez-Hernández VV (2021). The connection of the clinical learning environment and supervision of nursing students with student satisfaction and future intention to work in clinical placement hospitals. J Clin Nurs.

[42] Salminen L, Tuukkanen M, Clever K, Fuster P, Kelly M, Kielé V, Koskinen S, Sveinsdóttir H, Löyttyniemi E, Leino-Kilpi H, PROCOMPNurse-Consortium (2021). The competence of nurse educators and graduating nurse students. Nurse Educ Today.

[43] von Elm E, Altman DG, Egger M, Pocock SJ, Gøtzsche PC, Vandenbroucke JP, Initiative STROBE (2014). The strengthening the reporting of observational studies in epidemiology (STROBE) statement: guidelines for reporting observational studies. Int J Surg.

[44] Koskinen S, Pajakoski E, Fuster P, Ingadottir B, Löyttyniemi E, Numminen O, Salminen L, Scott PA, Stubner J, Truš M, Leino-Kilpi H, ProCompNurse Consortium (2021). Analysis of graduating nursing students’ moral courage in six European countries. Nurs Ethics.

[45] Sousa VD, Rojjanasrirat W (2011). Translation, adaptation and validation of instruments or scales for use in cross-cultural health care research: a clear and user-friendly guideline. J Eval Clin Pract.

[46] World Medical Association (2013). World Medical Association Declaration of Helsinki. Ethical principles for medical research involving human subjects. JAMA.

[47] All European Academies, ALLEA (2017). The European Code of Conduct for Research Integrity. Revised edition.

[48] Regulation (EU) 2016/679 on the protection of natural persons with regard to the processing of personal data and on the free movement of such data. The European Parliament and the Council of the European Union; 2016. http://data.europa.eu/eli/reg/2016/679/oj. Accessed 21 Oct 2021.

[49] Sveinsdóttir H, Gunnarsdóttir ÞJ, Björnsdóttir K, Hafsteinsdóttir TB, Jónsdóttir H, Kirkevold M, Leino-Kilpi H, Lomborg K, Rahm Hallberg I (2019). Towards the future: the education of nurses in Iceland reconsidered. Leadership in nursing: experiences from the European Nordic countries.

[50] Nursing and Midwifery Board of Ireland, NMBI (2018). Nurse registration programmes standards and requirements.

[51] Vega CMS, Espinosa AM, Alonso CL, Alonso SC, Martínez GA, Riesgo VCL (2020). Una fotografía de la imagen social de la Enfermería. RqR Enfermería Comunitaria.

[52] Gobierno de España. Real Decreto 592/2014, de 11 de julio, por el que se regulan las prácticas académicas externas de los estudiantes universitarios: 60502-60511. 2014. https://www.boe.es/buscar/pdf/2014/BOE-A-2014-8138-consolidado.pdf. Accessed 21 Oct 2021.

[53] Rafferty AM, Busse R, Zander-Jentsch B (2019). Strengthening health systems through nursing: evidence from 14 European countries.

[54] Sander M, Loos S, Temizdemir E (2019). Analyse der Situation der Pflege und Geburtshilfe (Hebammen) in den Münchner Krankenhäusern.

[55] Vereinte Dienstleistungsgewerkschaft. Ausbildungsreport Pflegeberufe 2015. http://jugend.dgb.de/++co++25e23860-f717-11e5-a09b-525400808b5c/Ausbildungsreport-Pflegeberufe-2015-der-verdi-Jugend.pdf. Accessed 21 Oct 2021.

[56] Völkel-Söte C, Martach D. Feindseligkeit in der Pflegeausbildung. “Bist du blöd, oder was?”. Die Schwester Der Pfleger. 2016;8 https://www.bibliomed-pflege.de/sp/artikel/23976-bist-du-bloed-oder-was. Accessed 21 Oct 2021.

[57] Pedregosa S, Fabrellas N, Risco E, Pereira M, Stefaniak M, Şenuzun F, Martin S, Zabalegui A (2021). Implementing dedicated education units in 6 European undergraduate nursing and midwifery students clinical placements. BMC Nurs.

[58] Ekstedt M, Lindblad M, Löfmark A (2019). Nursing students’ perception of the clinical learning environment and supervision in relation to two different supervision models – a comparative cross-sectional study. BMC Nurs.

[59] Cantrell MA, Lupinacci P (2007). Methodological issues in online data collection. J Adv Nurs.

